# On the Therapeutic Value of Sulphurous Acid in Scarlatina Maligna

**Published:** 1883-05

**Authors:** 


ON THE THERAPEUTIC VALUE OF SULPHUROUS ACID IN SCARLATINA MALIGNA.

Dr. Keith Norman Macdonald, after denying the prevalent opinion, that no reliance can be placed on any drug in cases of scarlatina, does not hesitate in affirming that, when properly applied, both locally and internally, sulphurous acid is by far the most efficacious remedy we possess. He continues, "I have had several opportunities of testing its efficacy in some of the worst cases I have ever seen, during the epidemic which has been rife in this town (Cupar Fife) for the last two months, and I am bound to say that, of all remedial measures in this disease, it is, in my opinion, the most reliable. My treatment is as follows: The moment the throat begins to become affected, I administer to a child, say of about six years of age, ten minims of the sulphurous acid, with a small quantity of glycerine in water, every two hours, and I direct the sulphurous acid spray to be applied every three hours to the fauces for a few minutes at a time, by using the pure acid, in severe cases, or equal parts of the acid and water, according to the severity of the case. Sulphur should also be burned in the sick chamber half a dozen times a day, by placing flour of sulphur upon a red hot cinder, and diffusing the sulphurous acid vapor through the room, until the atmosphere begins to become unpleasant to breathe.

"In the worst cases, where medicine cannot be swallowed, this and the spray must be entirely relied upon; and the dark shades which collect upon the teeth and lips should be frequently laved with a solution of the liquor potass. permanganatis of the strength of about one drachm to six ounces of water, some of which should be swallowed if possible.

"In cases presenting a diphtheritic character, the tincture of perchloride of iron should be administered in rather large doses in a separate mixture with chlorate of potash, and equal parts of the same with glycerine should be applied locally, with a camel's hair brush several times in the day; but, as in the majority of cases among children, it is next to impossible to use a local application more than once, the spray and permanganate solution will then prove of great service.

"As to other remedies recommended by various authors, ammonia is nasty, and cannot be taken well by children; carbolic acid has the same fault, and cannot be applied properly. Gargles are also useless in children, because they seldom reach the diseased surfaces, and warm baths and wet sheet packing are dangerous, because they are never carried out properly in private practice. The hypodermic injection of pilocarpine is a remedy that may give good results hereafter, but I have had no experience of its use."—*British Medical Journal.*

